# Supplementary material for: IoT in Water Quality Monitoring—Are We Really Here?
Source: Sensors (Basel). 2023 Jan 14;23(2):960. doi: 10.3390/s23020960 (PMC9864729; doi:10.3390/s23020960)
Supplement: Supplementary file 1 [file sensors-23-00960-s001.zip › Code S1 Python.pdf]

Code S1 Python

```
import time
import serial

# Set up the serial port for communication with the pH, Eh, and oxygen electrode
ser = serial.Serial('/dev/ttyUSB0', 9600, timeout=1)

# Send a command to the electrode to start measuring pH
ser.write(b'MEASURE:PH\n')

# Wait for the measurement to be taken
time.sleep(1)

# Read the measurement from the serial port
response = ser.readline().strip()

# Parse the measurement value from the response
pH = float(response.split(':')[1])

# Print the measurement value
print('pH:', pH)

# Send a command to the electrode to start measuring Eh
ser.write(b'MEASURE:EH\n')

# Wait for the measurement to be taken
time.sleep(1)

# Read the measurement from the serial port
response = ser.readline().strip()

# Parse the measurement value from the response
Eh = float(response.split(':')[1])

# Print the measurement value
print('Eh:', Eh)

# Send a command to the electrode to start measuring oxygen
ser.write(b'MEASURE:OXYGEN\n')

# Wait for the measurement to be taken
time.sleep(1)

# Read the measurement from the serial port
response = ser.readline().strip()
```

```
# Parse the measurement value from the response
oxygen = float(response.split(':')[1])

# Print the measurement value
print('Oxygen:', oxygen)

# Close the serial port
ser.close()
```

when the button is pressed, it reads the sensor data and prints it to the console.

This code assumes that the pH, Eh, and oxygen electrode is connected to the Raspberry Pi via a USB-to-serial adapter, and that the serial port is located at '/dev/ttyUSB0'. It also assumes that the electrode uses a specific protocol for communicating with the Raspberry Pi, in which commands and measurement values are sent over the serial port as ASCII strings. You may need to modify the code to match the specific protocol and communication settings of your electrode.

To configure a Raspberry Pi using Python, you can use the built-in configparser module to read and write configuration settings from a configuration file. Here's an example of how you might use this module to configure your Raspberry Pi:

```
import configparser

# Create a new ConfigParser object
config = configparser.ConfigParser()

# Read in the configuration file
config.read('config.ini')

# Set a new configuration option
config.set('Section1', 'option1', 'value1')

# Save the configuration file
with open('config.ini', 'w') as configfile:
    config.write(configfile)
```

This code reads in a configuration file called config.ini, sets a new option called option1 in the Section1 section, and then saves the updated configuration file. The configuration file is in the INI format, which consists of sections that contain options and their corresponding values.

You can also use the subprocess module to run shell commands on the Raspberry Pi, allowing you to perform various configuration tasks such as installing packages or modifying system settings. Here's an example of how you might use this module to install the numpy package:

```
import subprocess
```

```
# Install the numpy package using pip
subprocess.run(['pip', 'install', 'numpy'])
```

This code runs the pip install numpy command using the subprocess.run() function, which installs the numpy package for use in your Python programs.

You can find more information about the configparser and subprocess modules in the Python documentation:

https://docs.python.org/3/library/configparser.html
https://docs.python.org/3/library/subprocess.html
