# Supplementary material for: IoT in Water Quality Monitoring—Are We Really Here?
Source: Sensors (Basel). 2023 Jan 14;23(2):960. doi: 10.3390/s23020960 (PMC9864729; doi:10.3390/s23020960)
Supplement: Supplementary file 1 [file sensors-23-00960-s001.zip › Code S2 Julia.pdf]

Code S2 Julia

To install Julia on a Raspberry Pi, you can follow these steps:

Update the package manager's package list by running the following command:

```
sudo apt update
```

Install the Julia package using the package manager:

```
sudo apt install julia
```

Once Julia is installed, you can start using it by running the julia command in the terminal. This will open the Julia REPL (Read-Eval-Print Loop), which allows you to enter and execute Julia commands interactively.

You can also write Julia programs in a text editor and run them using the julia command followed by the name of the script file. For example:

```
julia my_program.jl
```

There are many resources available online for learning Julia, including the official Julia documentation and tutorials, as well as online courses and books. You can find more information about Julia at the following links:

https://julialang.org/
https://docs.julialang.org/
https://julialang.org/learning/

To configure a Raspberry Pi using the Julia programming language, you can use Julia's built-in JSON and FileIO modules to read and write configuration settings from a configuration file. Here's an example of how you might use these modules to configure your Raspberry Pi:

```
using JSON, FileIO

# Read in the configuration file
config = JSON.parsefile("config.json")

# Modify a configuration option
config["option1"] = "value1"

# Save the configuration file
open("config.json", "w") do io
```

```
    JSON.print(io, config)
end
```

This code reads in a configuration file called config.json, modifies the value of the option1 option, and then saves the updated configuration file. The configuration file is in the JSON format, which consists of key-value pairs.

Here is an example of Julia code that can be used to connect a Raspberry Pi to a pH, Eh, and oxygen electrode:

```
using SerialPorts, DelimitedFiles

# Set up the serial port for communication with the pH, Eh, and oxygen electrode
ser = SerialPort("/dev/ttyUSB0", 9600)

# Send a command to the electrode to start measuring pH
write(ser, "MEASURE:PH\n")

# Read the measurement from the serial port
response = readline(ser)

# Parse the measurement value from the response
pH = split(response, ':')[2] |> parse

# Print the measurement value
println("pH: $pH")

# Send a command to the electrode to start measuring Eh
write(ser, "MEASURE:EH\n")

# Read the measurement from the serial port
response = readline(ser)

# Parse the measurement value from the response
Eh = split(response, ':')[2] |> parse

# Print the measurement value
println("Eh: $Eh")

# Send a command to the electrode to start measuring oxygen
write(ser, "MEASURE:OXYGEN\n")

# Read the measurement from the serial port
response = readline(ser)

# Parse the measurement value from the response
```

```
oxygen = split(response, ':')[2] |> parse

# Print the measurement value
println("Oxygen: $oxygen")

# Close the serial port
close(ser)
```

This code assumes that the pH, Eh, and oxygen electrode is connected to the Raspberry Pi via a USB-to-serial adapter, and that the serial port is located at '/dev/ttyUSB0'. It also assumes that the electrode uses a specific protocol for communicating with the Raspberry Pi, in which commands and measurement values are sent over the serial port as ASCII strings. You may need to modify the code to match the specific protocol and communication settings of your electrode.
