# Supplementary material for: IoT in Water Quality Monitoring—Are We Really Here?
Source: Sensors (Basel). 2023 Jan 14;23(2):960. doi: 10.3390/s23020960 (PMC9864729; doi:10.3390/s23020960)
Supplement: Supplementary file 1 [file sensors-23-00960-s001.zip › Table S1.pdf]

Table S1

IoT	Short description	Examples	Examples from the market
Sensors	A device that is used to measure a physical or chemical indices. Need to be connected to a meter or device which will connect to IoT system.	pH sensors, Eh, sensors, COD sensors, temperature sensor, N-NH_4^+ sensors, turbidity sensors, TDS sensors	1. M-Node Smart Sensors [38]<br>2. Water sensors from YSI [39]<br>3. Online-sensors by Hach [40]
Smart metres	A Device which can made computations after receiving data from specific sensor and may perform a decision what to do. Can connect to IoT system.	Water usage meter<br>Water management meters – monitors the water quality, pressure	1. ChloroNet<br>2. SeptiNet<br>3. MetriNet<br>4. Online analyzers by Hach [40]
Actuators	An actuator is a machine component or system that moves or controls the mechanism of the system. Perform coded decision.	Hydraulic Actuators<br>Pneumatic Actuators<br>Electrical Actuators<br>Thermal/Magnetic Actuators<br>Mechanical Actuators	1. Belimo series [41]<br>2. Tuya solutions [42]<br>3. Auma solutions [43]
Gateways	An IoT Gateway is a solution for enabling IoT communication, usually device -to-device communications or device-to-cloud communications.	An IoT gateway for water quality monitoring could be used to collect data from sensors that measure various parameters of water quality, such as pH, temperature, conductivity, and dissolved oxygen. The gateway could then transmit this data to a cloud platform or other central location, where it could be analyzed and used to monitor the health of bodies of water or to identify potential pollution sources. The gateway could also be programmed to send alerts or notifications if certain thresholds are exceeded, such as if the water temperature becomes too high or the pH falls outside of a certain range. The gateway could be solar-powered or connected to a grid to allow for continuous monitoring	1. Siemens [44]<br>2. Intel [45]

References

38. Smart sensor, data rich technology. Available online: https://www.analyticaltechnology.com/us/water-monitoring/smart-water-solutions/ (accessed on 04.01.2023)
39. Water Sensors and Water Monitoring Equipment. Available online: https://www.ysi.com/products (accessed on 04.01.2023).
40. Ensuring Water Quality for People Around the World. Available online: https://hach.com (accessed on 04.01.2023).
41. IoT Actuators. Available online: https://www.belimo.com/us/shop/es_US/Systems/IoT-Actuators/c/17707-18867 (accessed on 04.01.2023).
42. Available online: https://solution.tuya.com (accessed on 04.01.2023).
43. Multi-turn Actuators. Available online: http://auma.polinski.com.pl/products/multi-turn-actuators-auma/ (accessed on 04.01.2023).
44. SIMATIC IOT gateways. Available online: https://new.siemens.com/global/en/products/automation/pc-based/iot-gateways.html (accessed on 04.01.2023).
45. Intel IoT Gateway Technology. Available online: https://www.intel.pl/content/www/pl/pl/embedded/solutions/iot-gateway/training/iot-gateway-training-overview.html (accessed on 04.01.2023).
